# Vulnerabilities to Temperature Effects on Acute Myocardial Infarction Hospital Admissions in South Korea

**DOI:** 10.3390/ijerph121114571

**Published:** 2015-11-13

**Authors:** Bo Yeon Kwon, Eunil Lee, Suji Lee, Seulkee Heo, Kyunghee Jo, Jinsun Kim, Man Sik Park

**Affiliations:** 1 Department of Public Health, Graduate School, Korea University, 73, Inchon-ro, Seongbuk-gu, Seoul 02841, Korea; E-Mails: boyeon02@gmail.com (B.Y.K.); seulkeeheo@naver.com (S.H.); 2 Department of Preventive Medicine, College of Medicine, Korea University, 73, Inchon-ro, Seongbuk-gu, Seoul 02841, Korea; 3 Graduate School of Public Health, Graduate School, Korea University, 73, Inchon-ro, Seongbuk-gu, Seoul 02841, Korea; E-Mails: jkh861114@nate.com (K.J.); lilykim1011@gmail.com (J.K.); 4 Department of Statistics, College of Natural Science, Sungshin Women’s University, 249-1, Dongseon-dong 3-ga, Seongbuk-gu, Seoul 02844, Korea; E-Mail: mansikpark@sungshin.ac.kr

**Keywords:** myocardial infarction, hospital admissions, temperature, socioeconomic status, Medicaid, gender, age

## Abstract

Most previous studies have focused on the association between acute myocardial function (AMI) and temperature by gender and age. Recently, however, concern has also arisen about those most susceptible to the effects of temperature according to socioeconomic status (SES). The objective of this study was to determine the effect of heat and cold on hospital admissions for AMI by subpopulations (gender, age, living area, and individual SES) in South Korea. The Korea National Health Insurance (KNHI) database was used to examine the effect of heat and cold on hospital admissions for AMI during 2004–2012. We analyzed the increase in AMI hospital admissions both above and below a threshold temperature using Poisson generalized additive models (GAMs) for hot, cold, and warm weather. The Medicaid group, the lowest SES group, had a significantly higher RR of 1.37 (95% CI: 1.07–1.76) for heat and 1.11 (95% CI: 1.04–1.20) for cold among subgroups, while also showing distinctly higher risk curves than NHI for both hot and cold weather. In additions, females, older age group, and those living in urban areas had higher risks from hot and cold temperatures than males, younger age group, and those living in rural areas.

## 1. Introduction

Scientists have suggested that most of the human health effects of climate change will be adverse [1,2]. The Intergovernmental Panel on Climate Change (IPCC) has also highlighted the many effects of temperature variations on human health, including the effects on temperature-sensitive chronic diseases [3]. Among cardiovascular diseases, the likely direct impact is on acute myocardial infarction (AMI), a major cause of death and disability worldwide [4,5], because winter weather and heat waves primarily cause death by AMI [6]. Dilaveris *et al.* also reported that ambient temperature is an important predictor of AMI mortality [7].

Several studies have shown the effect of both high and low temperatures on AMI hospital admissions [8,9,10,11,12] and mortality [7,13,14,15]. Bhaskaran *et al.*, in an extensive review of prior studies, showed that both hot and cold weather had negative effects on the short-term AMI risk [16]. Cheng and Su reported that increased physical stress caused by heat and cold was related to an increase in blood pressure and vasoconstriction, which may lead to AMI [17]. Moreover, a study in South Korea demonstrated that the threshold temperature differed according to geographic location as well as the risk of AMI occurrence shown during both heat and cold exposure at threshold temperatures [18].

An increased susceptibility to temperature according to demographic population factors such as gender and age has become an issue of interest in the scientific community [19,20,21]. A number of studies have shown that the elderly and females were the populations most susceptible to temperature-associated AMI [9,10,13,15]. In contrast, in Copenhagen (Denmark) the strongest association between temperature and AMI hospital admissions was observed in males and younger age (19–65) groups [8]. Further, Wichmann *et al.* reported that no susceptible groups were identified based on age or sex [22].

Most previous studies have evaluated susceptible populations according to age and gender, while data on vulnerability factors related to population density (living area) and individual socioeconomic status (SES) remain unclear or inadequate [20,23,24]. Living in densely populated urban areas was an important risk factor, with many individuals vulnerable to adverse heat-related health outcomes because of the urban heat island effect [25]. However, Hattis *et al.* reported demographic characteristics such as age and ethnicity were more important factors than urbanization [26]. Most have studies focused on mortality due to urban heat effects, with lack of evidence for cold effects.

Recently, socioeconomic factors (e.g., education and income) have also been considered as an important component of vulnerability to temperature-associated mortality [23]. Among the elderly, those with underlying cardiovascular disease (CVD), and those who are poor, uneducated, or isolated begin to experience a rapid rise in mortality in extreme weather because of their lack of access to educational material and information resources, as well as their impaired physiological responsiveness [27,28]. Low SES status has been found to compound the relationship between temperature and mortality [29,30]. Moreover, low SES may be associated with inequality of adaptation or ability to mitigate responses to climate change [31,32]. A few studies have shown individual SES is independently and significantly associated with the incidence of AMI [33,34], while little is known about the effect of temperature on AMI hospital admissions or mortality by individual SES.

The objective of this study was to determine the associations between temperature and AMI hospital admissions by subpopulation (gender, age, living area, and SES) using the Korea National Health Insurance (KNHI) database, which covers the entire South Korean population [35]. The seasons were divided into hot, cold, and warm weather periods to determine the differences in heat and cold effects at extreme and mild temperatures. We aimed to determine the association between threshold temperatures and relative risk (RR) for vulnerability to heat and cold on AMI hospital admissions in each weather period, according to living area (urban or rural) and individual SES among subpopulations.

## 2. Materials and Methods

### 2.1. Study Area

The study area was South Korea (latitude is 37°00′ N and longitude is 127°30′ E), located in the southern part of the Korean peninsula in Eastern Asia. South Korea has an area of 99,720 square km (38,502 square miles) and a 2413 km (1499 mile)-long coastline [36,37]. The population of South Korea in July 2014 was estimated at about 50 million [38]. There are four seasons in a year in South Korea, according to Meteorological Administration climate data, with characteristics as follows [39]: Summer (June–August) shows high temperatures and humidity under the influence of the North Pacific high pressure system; winters (December–February) are cold and dry under the influence of continental high pressure systems; spring (March–May) and autumn (September–November) are often clear and dry due to the impact of migratory anticyclones.

### 2.2. Health Outcome Data

We obtained daily AMI hospital admission data from the Korea National Health Insurance Corporation (KNHIC) covering the period from 1 January 2004 to 31 December 2012. The primary diagnosis code of AMI was I210–214 and 219 according to the International Classification of Diseases 10th revision (ICD-10). We excluded AMI hospital admissions that occurred within 30 days after a previous AMI hospital admission because readmissions following discharge for AMI within a month are regarded as recurrences [40]. We divided vulnerable populations by individual SES according to enrollment in National Health Insurance (NHI) or medical assistance (Medicaid). NHI covers about 97% of the general Korean population; the other 3% in low-income brackets are covered by Medicaid [41]. Moreover, health insurance premiums for NHI are also used as a surrogate for income in South Korea [42]. We classified insurance premiums into three groups of low, medium, or high. Living area was divided based on population density. Cities with 500,000 or more residents were considered urban areas and counties with less than 500,000 residents were considered rural areas based on data from the Ministry of Security and Public Administration (MOSPA) in South Korea [43].

### 2.3. Climatological Data and Air Pollution Data

The climatological and air pollution data for 1 January 2004 to 31 December 2012 were used to assess the health outcomes for the same period. We obtained daily meteorological data from the Korea Meteorological Administration, including mean, maximum, and minimum temperatures (°C), relative humidity (%), precipitation, and sea-level pressure. These elements were observed at 68 weather stations. The air pollution data, including particulate matter (PM10), nitrogen dioxide (NO_2_), sulfur dioxide (SO_2_), carbon monoxide (CO), and ozone (O_3_) were obtained from the National Institute of Environmental Research. Daily air pollution monitoring was conducted at 238 sites located based on population density and covering all regions of South Korea.

### 2.4. Statistical Analysis

Most studies have used threshold temperatures based on percentiles such as the interquartile range (IQR) in study periods [8,14,44], but did not estimate the actual threshold temperatures that would show an increase in the risk of AMI. However, Lee *et al.* reported that piecewise regression (PR) analysis performed after plotting temperature and daily hospital admissions would be a better approach for identifying the threshold temperature [18]. We used threshold temperatures based on piecewise regression (PR) analysis to determine the inflection point of the relationship between daily adjusted hospital admissions and temperature by maximum R^2^.

We explored the effects of air temperature on AMI hospital admissions in hot (June–August), cold (December–February), and warm (March–May and September–November) weather periods. The thresholds for mean and maximum temperatures were used for determining the effect of heat in summer, while the thresholds for mean and minimum temperatures were used for assessing the effect of cold in winter. The warm period, corresponding to the change of seasons between summer and winter, generally had a moderate climate; therefore, we estimated the mean thresholds for heat and cold.

We also used generalized additive models (GAMs) analysis with smoothing spline functions to describe nonlinear relationships for time-series. We considered potential confounders such as long-term trends using the calendar year and the day of the week because hospital admissions have unique day patterns, with the highest numbers occurring on Monday and the lowest numbers observed on Saturday, Sunday, and holidays. The relationships were adjusted for humidity, sea-level pressure, amount of air pollutants (PM_10_, O_3_, and NO_2_) using the spline function, and the annual population size was considered as the offset variable in GAMs. In addition, we added the duration of a heat or cold wave into the GAMs for evaluation of the effects of extreme weather events in hot and cold weather periods. In South Korea, a heat wave is defined the heat index (HI) exceeds 41 °C using the maximum temperature and humidity, while a cold wave is defined by a daily minimum temperature of under −12 °C [39]. The relative risk (RR) were calculated for AMI hospital admissions per 1 °C above the mean and maximum threshold temperatures for heat as well as per 1 °C below the mean and minimum threshold temperatures for cold. To compare the RR for different subgroups, we used the same threshold temperatures.

Moreover, the effects of cold have been reported to be associated with delays, therefore the remaining terms covered delays of up to 28 days with weekly groupings chosen to allow more precise estimations of the effect and because at longer lags, any temperature effects would be unlikely to vary considerably from day to day [10]. Therefore, we analyzed risks for lags of 0, 1, 2–3, 4–7, 8–14, 15–21, and 22–28 days, respectively, in hot and cold weather. Lag was defined as the effect of the current day; lag 1 was the temperature on the prior 1 day, and lag X-Y was the average of prior X and Y days. However, in warm weather we analyzed risks for lags up to 6 days. We did not observe long-term lag effects in warm weather because of unpredictable weather during those seasons.

To confirm the general relationship between daily AMI hospital admissions and mean temperature (lag0), the smoothing spline was used with three degrees of freedom (df) for the confounding factors, such as day of the week, precipitation, humidity, sea-level pressure, air pollutants (PM_10_, O_3_, NO_2_), and annual population size by subgroups. We also used piecewise regression (PR) analysis to confirm the threshold temperature when the group showed distinctly higher risk curves.

We analyzed and compared the risks among several subpopulations in each period (hot, cold, or warm) by gender, age (20–74 or ≥75 years), living area (urban or rural), and individual SES. The SES was based on type of health insurance (NHI or Medicaid) and insurance premium (low, medium, or high). Data were analyzed using the statistical software SAS version 9.3 for Windows (SAS Institute Inc., Cary, NC, USA).

## 3. Results

### 3.1. Study Subjects and Meteorological Conditions

Our study subjects were a total of 179,099 AMI hospital admission patients between January 2004 and December 2012 in South Korea (Table 1). The mean age of AMI patients was 64.5 years. The percentage of AMI patients who were male, covered by NHI, and living in urban areas was higher compared to the other subgroups. The insurance premium showed similar ratios between low, medium, and high groups. However, the rates were calculated as the number of patients divided by the total population in each group for the study period. The AMI patients in Medicaid (8.7 per million people) showed higher rates than those in the NHI group (3.8 per million people).

Table 2 shows seasonal summary statistics for meteorological and air pollutants during the study period. The mean of maximum temperature in the hot weather was 28.56 °C and the mean of minimum temperature was −3.81 °C in the cold weather. The hot weather period had very high precipitation and humidity, while the cold weather period was dry, with low precipitation and humidity. The cold weather period had slightly higher air pollutant concentrations than other periods. The warm weather period had mild meteorological ranges.

### 3.2. The Vulnerability to Heat and Cold by Gender, Age and Area

Table 3 shows the relative risk (RR) of heat and cold for AMI hospital admissions both above and below a threshold temperature in hot and cold weather by gender, age, and living area, respectively. In hot weather, the RRs of temperature increases of 1 °C above the threshold of the mean temperature (28.5 °C) were evaluated for each subgroup. The ≥75 years old and urban area subgroups had high RRs of 1.16 (95% CI: 1.01–1.33) and 1.10 (95% CI: 1.02–1.20) for heat. The risk of AMI by gender was observed for heat exposure not statistically significant above mean threshold temperature. However, females showed a significantly higher RR than males, at 1.10 (95% CI: 1.01–1.20) above the maximum threshold temperature (33.5 °C) (Table S1). In cold weather, RRs for temperature decreases of 1 °C below the minimum temperature threshold (−13.5 °C) were examined for each subgroup. Females and urban dwellers showed significant high RRs of 1.05 (95% CI: 1.02–1.10) and 1.05 (95% CI: 1.01–1.10), respectively, for cold.

Table 4 shows the RRs of a temperature increase of 1 °C above the threshold of the mean temperature (18.5 °C) as well as for decreases of 1 °C below the threshold of the mean temperature (−0.5 °C) in warm weather. There was no overall population effect, and only females had a statistically significant high RR of 1.03 (95% CI: 1.01–1.05) for heat and 1.07 (95% CI: 1.01–1.12) for cold, respectively, among the other groups.

**Table 1:** Patients Characteristics of the acute myocardial infarction hospital admissions in South Korea during study period (1 January 2004–31 December 2012).

	Study Period	Hot Weather	Cold Weather	Warm Weather	*p*-Value
**No. of Patients**	179,099	42,831	45,428	90,840	
**Age (mean ± SD)**	64.53 ± 13.25	64.22 ± 13.26	64.66 ± 13.19	64.61 ± 13.26	
**Gender**					
Male	119,037 (66.46)	28,789 (67.22)	30,012 (66.06)	60,236 (66.31)	<0.0001
Female	60,062 (33.54)	14,042 (32.78)	15,416 (33.94)	30,604 (33.69)	
**Insurance type**					
NHI	165,276 (92.28)	39,481 (92.18)	41,749 (91.9)	84,046 (92.52)	<0.0001
Medicaid	13,823 (7.72)	3350 (7.82)	3679 (8.1)	6794 (7.48)	
**Insurance premium ^a^**					
Low	59,108 (33.00)	14,044 (32.79)	15,381 (33.86)	29,683 (32.68)	<0.0001
Medium	54,329 (30.33)	12,957 (30.25)	13,940 (30.69)	27,432 (30.20)	
High	65,662 (36.66)	15,830 (36.96)	16,107 (35.46)	33,725 (37.13)	
**Area ^b^**					
Urban	110,382 (61.63)	26,200 (61.17)	28,416 (62.55)	55,766 (61.39)	<0.0001
Rural	68,717 (38.37)	16,631 (38.83)	17,012 (37.45)	35,074 (38.61)	

Hot weather (summer): June–August; Cold weather (winter): December–February; Warm weather (spring and autumn): March–May and September–November; NHI: National health Insurance for general populations; Medicaid: Public health care benefits for the poor; ^a^ Insurance premium for NHI was calculate base on the value of properties and household income; ^b^ Area was divided base on registered population. Urban area with more than 500,000 and rural area with less than 500,000.

### 3.3. The Vulnerability to Heat and Cold by SES

Table 5 shows the effect of temperature on AMI hospital admissions by SES based on insurance type (NHI and Medicaid) and premium (low, medium, or high) in hot and cold weather. The Medicaid group was the most vulnerable, with the highest RR of 1.37 (95% CI: 1.07–1.76) for heat and 1.11 (95% CI: 1.04–1.20) for cold. The low insurance premium group also showed an increased risk of AMI hospital admissions for heat, but we did not observe any significant difference among insurance premiums for cold. The RR below the mean temperature threshold (−8.5 °C) showed a similar pattern (Table S2).

**Table 2:** Meteorological conditions and air pollution levels in South Korea during study period (1 January 2004–31 December 2012).

	Hot Weather				Cold Weather				Warm Weather			
	Mean	Range	Median	IQR	Mean	Range	Median	IQR	Mean	Range	Median	IQR
**Temperature**												
mean	24.19 ± 2.87	10.2–32.74	24.2	4.1	0.55 ± 4.71	−18.9–18.9	0.6	6.4	13.51 ± 6.30	−8.20–29.60	14.1	9.6
maximum	28.56 ± 3.5	11.4–38.7	28.8	4.9	5.54 ± 5.03	−11.5–24.5	5.5	7	19.02 ± 6.58	−4.50–35.90	19.7	9.8
minimum	20.74 ± 3.2	0.5–29.1	21	4.5	−3.81 ± 5.16	−27.7–16.1	−3.7	6.8	8.75 ± 6.78	−17.7–27.2	9.1	10.2
**Other meteorological variables**												
Precipitation (mm)	8.98 ± 24.18	0–449.5	0	5.0	0.93 ± 4.01	0–106	0.0	0.0	2.80 ± 10.44	0–420.0	0	0.2
Relative humidity (%)	75.59 ± 11.36	22.3–100	76.5	15.1	58.52 ± 15.75	11.3–100	58.9	22.9	63.51 ± 15.02	12.1–100	64.5	21.7
Sea-level pressure (hPa)	1007.7 ± 4.2	988.4–1021.0	1007.9	5.9	1023.6 ± 5.2	995.1–1039.5	1023.9	6.8	1016.51 ± 6.15	990.5–1034.9	1016.8	8.4
**Air pollutant levels**												
PM10 (μg/m^3^)	42.39 ± 21.86	1.5–249.21	38.43	27.07	56.77 ± 28.26	0–368.77	50.31	32.85	55.76 ± 37.72	2.54–1106.04	48.46	32.88
NO_2_ (ppm)	0.02 ± 0.01	0–0.1	0.02	0.01	0.03 ± 0.01	0–0.1	0.03	0.02	0.02 ± 0.01	0–0.11	0.02	0.01
CO (ppm)	0.43 ± 0.16	0–2.64	0.41	0.19	0.78 ± 0.39	0–6.03	0.7	0.46	0.57 ± 0.24	0–3.32	0.52	0.26
SO_2_ (ppb)	4.14 ± 2.21	0–42.3	3.7	2.3	7.58 ± 4.09	0–60.1	6.7	4.2	5.22 ± 2.69	1.5–35.2	4.7	2.9
O_3_ (ppm)	0.03 ± 0.01	0–0.09	0.02	0.02	0.02 ± 0.01	0–0.09	0.02	0.01	0.03 ± 0.01	0–0.09	0.02	0.02

Hot Weather (summer): June–August; Cold Weather (winter): December–February; Warm Weather (spring and autumn): March–May and September–November; IQR: interquartile range.

**Table 3:** The relative risk (RR) of heat and cold effects on AMI hospital admissions in hot and cold weather period by gender, age, and area.

	Heat Effect ^a^		Cold Effect ^b^	
	Lag ^c^	RR (95% CI)	Lag ^c^	RR (95% CI)
**All**	0	1.05 (0.98–1.12)	2–3	1.03 (1.01–1.05) *
**Gender**				
Male	0	1.05 (0.96–1.14)	2–3	1.03 (1.00–1.05)
Female	0	1.05 (0.93–1.18)	1	1.05 (1.02–1.10) *
**Age**				
20–74 years	0	1.01 (0.94–1.10)	2–3	1.03 (1.00–1.05)
≥75 years	0	1.16 (1.01–1.33) *	2–3	1.04 (1.00–1.08)
**Area**				
Urban	0	1.10 (1.02–1.20) *	2–3	1.05 (1.01–1.10) *
Rural	1	1.03 (0.95–1.12)	2–3	1.03 (1.01–1.06) *

CI: Confidence interval; Models adjusted for calendar year, day of the week, precipitation, humidity, sea-level pressure, and air pollutants (PM_10_, O_3_, NO_2_) as spline functions and considering the annual population size as the offset variable; ^a^ Temperature increase of 1 °C above the threshold mean temperature (28.5 °C) in hot weather (June–August). Considering duration of heat wave in model; ^b^ Temperature decrease of 1 °C below the threshold minimum temperature (−13.5 °C) in cold weather (December–February). Considering duration of cold wave in model; ^c^ Lags of 0, 1, 2–3, 4–7, 8–14, 15–21, 22–28; * *p* < 0.05.

**Table 4:** The relative risk(RR) of heat and cold effects on AMI hospital admissons in warm weather period by gender, age, and area.

	Heat Effect ^a^		Cold Effect ^b^	
	Lag ^c^	RR (95% CI)	Lag ^c^	RR (95% CI)
**All**	4	1.01 (1.00–1.02)	4	1.02 (0.98–1.06)
**Gender**				
Male	2	1.01 (0.99–1.02)	5	1.03 (0.98–1.08)
Female	4	1.03 (1.01–1.05) *	2	1.07 (1.01–1.12) *
**Age**				
20–74 years	4	1.01 (0.99–1.02)	5	1.03 (0.98–1.08)
≥75 years	4	1.02 (1.00–1.05)	2	1.06 (0.99–1.13)
**Area**				
Urban	4	1.01 (1.00–1.02)	2	1.02 (0.97–1.06)
Rural	1	1.01 (0.99–1.03)	4	1.03 (0.97–1.10)

CI: Confidence interval; Models adjusted for calendar year, day of the week, precipitation, humidity, sea-level pressure, and air pollutants (PM_10_, O_3_, NO_2_) as spline functions and considering the annual population size as the offset variable; ^a^ Temperature increase of 1 °C above the threshold mean temperature (18.5 °C) in warm weather (March–May and September–November); ^b^ Temperature decrease of 1 °C below the threshold mean temperature (−0.5 °C) in warm weather; ^c^ Lags up to 6 days; * *p* < 0.05.

**Table 5:** The relative risk (RR) of heat and cold effects on AMI hospital admissions in hot and cold weather period by insurance type and premium.

	Heat Effect ^a^		Cold Effect ^b^	
	Lag ^c^	RR (95% CI)	Lag ^c^	RR (95% CI)
**Insurance type**				
NHI	0	1.03 (0.96–1.11)	2–3	1.03 (1.00–1.05)
Medicaid	0	1.37 (1.07–1.76) *	2–3	1.11 (1.04–1.20) *
**Insurance premium**				
Low	0	1.14 (1.02–1.29) *	2–3	1.03 (1.00–1.07)
Medium	0	1.08 (0.95–1.22)	2–3	1.03 (0.99–1.07)
High	1	1.02 (0.94–1.09)	1	1.04 (1.00–1.09)

CI: Confidence interval; Models adjusted for calendar year, day of the week, precipitation, humidity, sea-level pressure, and air pollutants (PM10, O_3_, NO_2_) as spline functions and considering the annual population size as the offset variable; ^a^ Temperature increase of 1 °C above the threshold mean temperature (28.5 °C) in hot weather (June–August). Considering duration of heat wave in model; ^b^ Temperature decrease of 1 °C below the threshold minimum temperature (−13.5 °C) in cold weather (December–February). Considering duration of cold wave in model; ^c^ Lags of 0, 1, 2–3, 4–7, 8–14, 15–21, 22–28; * *p* < 0.05.

In warm weather (Table 6), the mean threshold temperature for heat was 18.5 °C and −0.5 °C for cold. We found that Medicaid had a high RR of 1.06 (95% CI: 1.01–1.10) for heat. Both Medicaid and low insurance premiums also showed a high RR of 1.10 (95% CI: 0.92–1.31) and 1.07 (95% CI: 1.01–1.15) for cold, respectively.

**Table 6:** The relative risk (RR) of heat and cold effects on AMI hospital admissions in warm weather period by insurance type and premium.

	Heat Effect ^a^		Cold Effect ^b^	
	Lag ^c^	RR (95% CI)	Lag ^c^	RR (95% CI)
**Insurance type**				
NHI	4	1.01 (1.00–1.02)	4	1.02 (0.98–1.06)
Medicaid	1	1.06 (1.01–1.10) *	5	1.10 (0.92–1.31)
**Insurance premium**				
Low	4	1.02 (1.00–1.04)	5	1.07 (1.01–1.15) *
Medium	6	1.01 (1.00–1.03)	4	1.05 (0.97–1.13)
High	1	1.01 (0.99–1.03)	6	1.02 (0.97–1.08)

CI: Confidence interval; Models adjusted for calendar year, day of the week, precipitation, humidity, sea-level pressure, and air pollutants (PM10, O_3_, NO_2_) as spline functions and considering the annual population size as the offset variable; ^a^ Temperature increase of 1 °C above the threshold mean temperature (18.5 °C) in warm weather (March–May and September–November); ^b^ Temperature decrease of 1 °C below the threshold mean temperature (−0.5 °C) in warm weather; ^c^ Lags up to 6 days; * *p* < 0.05.

**Figure 1:**
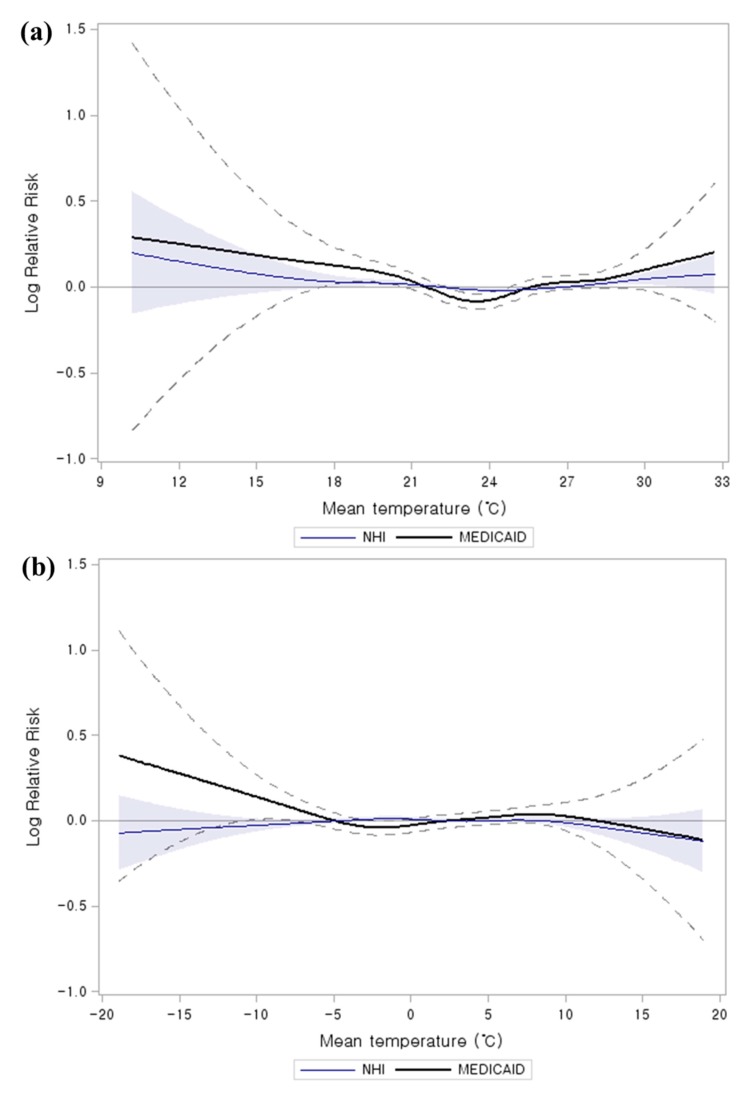
Comparison of Medicaid and NHI on the relationship between mean temperature (lag 0) and AMI hospital admissions. The curve shows smoothing spline of mean temperature with 3 degrees of freedom. The model was adjusted for calendar year, day of the week, precipitation, humidity, sea-level pressure, air pollutants (PM_10_, O_3_, NO_2_), and annual population size: (**a**) hot weather (summer: June–August) (**b**) cold weather (winter: December–February) (Blue areas and black dotted Upper and lower 95% confidence intervals).

We plotted the smoothing spline function in GAMs to compare of Medicaid and NHI on the relationship between mean temperature (lag 0) and AMI hospital admissions (Figure 1). The Medicaid group showed distinctly higher risk curves than NHI for both hot and cold weather. In addition, the threshold temperatures for AMI hospital admissions were strikingly different between the Medicaid and NHI groups in hot weather. The point increases in the risk of AMI hospital admissions using spline function was confirmed by PR analysis. The Medicaid group showed a lower threshold temperature of 23.5 °C as compared to for NHI groups (28.5 °C) in the hot weather.

## 4. Discussion

Temperature-associated AMI studies are usually done in both hot/warm and cold weather periods in each country [8,44,45]. However, we divided seasons according to hot (June to August), cold (December to February), and warm (March to May and September to November) weather periods to determine threshold temperatures, because South Korea has distinct seasons. Spring and fall have a generally moderate climate, while summer and winter have extreme weather events, such as heat and cold waves [46]. We determined 28.5 °C to be the threshold temperature for sudden increases in AMI hospital admissions due to heat in hot weather. Our threshold for heat-related effect for AMI was similar to that found in other countries, such as for Sao Paulo in Brazil (24 °C–27 °C) [47], and Melbourne in Australia (27 °C–30 °C) [48], as well as for another South Korean study (27.5 °C–28.5 °C) [18].

In cold weather periods, the thresholds were determined to be a mean temperature of −8.5 °C and a minimum temperature of −13.5 °C in this study. There have been similar mean threshold temperatures in cold regions, such as in the Czech Republic (−5.7 °C or −7.6 °C) [49] and in Russia (−6 °C) [50]. We also calculated the relative risk (RR) below the minimum threshold temperature, because these reflected the extreme low temperatures better than the mean temperature in cold weather, which tend to occur at irregular intervals of about 2–7 days in South Korea [51]. In warm weather, the threshold temperature was calculated for the effects of both heat and cold because temperatures tend to increase or decrease suddenly. Few studies have reported the threshold temperatures in warm seasons, as in our study. Therefore, we compared the threshold temperatures with those for similar temperature seasons in other countries. The threshold temperature for heat was 18.5 °C, and −0.5 °C for cold in our study. England and Wales showed an estimated threshold of 20 °C for heat in the warmest months (June to August) [12], and some studies showed a threshold for cold below 0 °C, such as −1.5 °C or −2.5 °C [18,52].

Our study evaluated susceptible populations by demographic characteristics, such as gender and age. Precious studies that examined the effects of temperature separately for different groups, have shown inconsistent results. Basu also reported that demographic characteristics (being female and elderly) can modify the severity of effects on temperature through various physiological and behavioral pathways [21]. Wichmann *et al.* reported that no susceptible groups were identified in Gothenburg (Sweden) based on age or gender [22], while males and 19–65 years seemed to be more susceptible to AMI hospital admissions because males had a higher risk of developing CVD than females, and those in the age group of 19–65 years participated in more outdoor activities in Copenhagen (Denmark) [8].

However, we determined that females had high risk of AMI hospital admission in cold periods. Females also showed significantly higher RRs above the maximum threshold temperature in hot weather. A risk for heat and cold was observed in females, which is consistent with prior confirmed outcomes [13,53,54]. This could be because females have a higher risk for arrhythmia, ischemia, and high blood pressure, all of which are more affected by extreme hot and cold temperatures [55].

Among age groups, we found that those ≥75 years showed susceptibility to heat. The effect of cold on those ≥75 years also showed a slightly higher RR than for those aged 20–74 years, but this was not statistically significant. There have been many studies reporting that the elderly were vulnerable both to heat [12,56] and cold [7,10,13,15]. Older people undergo physiological changes in renal function and electrolyte homeostasis in extremely hot weather [57], and have a weaker thermoregulation system due to reduced cutaneous thermal sensitivity and diminished skin vasoconstriction with cold stress [58].

We evaluated the association between temperature and AMI hospital admissions by population level (urban or rural area) and individual SES factors in South Korea. Our study showed that urban populations were more susceptible to AMI than rural populations in hot weather and similar risks in cold weather were found in both urban and rural areas. Urban *et al.* reported more pronounced effects of heat stress on CVD during warm days in urban areas, while stronger cold effects were observed in a rural population [49]. Hajat *et al.* also showed a greater heat effect on mortality among those living in urban areas, while cold effects were greater in some rural areas [20]. The heat effect in urban areas is probably due to the heat-island effect, whereby greater heat retention occurs in more heavily built-up areas of large cities, including in South Korea [59]. Our study did not show a higher risk in the rural population in cold weather, probably because of reduced daily activities in the winter season.

Most studies have reported that lower SES increased the temperature-associated mortality, with SES defined by low income [60], low education [24,61,62], or living in poverty [63]. Madrigano *et al.* reported that persons living in areas with greater poverty were more susceptible to heat-related AMI occurrence [44]. However, Wichmann *et al.* reported that high SES groups seemed to be more susceptible to cold-related AMI hospital admissions in Copenhagen. This might be because high SES groups live in owned detached houses, so that cold weather behaviors such as snow shoveling could be related to increased ischemic heart disease [8]. In this study, we focused on evaluating temperature-associated AMI hospital admissions by individual insurance type and premium based on the KNHI data, which covers the general Korean population. Therefore, this study was able to identify the effect of individual SES on increased AMI hospital admissions associated with temperature.

We observed that temperature-related AMI hospital admissions were more pronounced in low SES groups (Medicaid and low premium health insurance) for both heat and cold. The Medicaid population, especially the lowest SES group, had the highest significant RR among subgroups as well as confirmed distinctly higher risk curves both in hot and cold weather [22]. Most studies showed that persons in low SES groups tend to have inadequate means (e.g., for heating/cooling systems and clothing), live in residences constructed with poor quality materials, and have poor health status [25,60]. The low SES population lifestyle seemed to be more vulnerable to weather, which could affect increased AMI hospital admissions.

This study has several limitations. Based on its environmental design, individual exposure levels to meteorological conditions and air pollutants were estimated by area monitoring data. Individual medical history or personal behaviors were not reflected because of lack of information. We also would not have included pre-hospitalization fatal AMIs. However, we used the data from KNHI, which is the only insurance program that universally covers the Korean population. Despite these limitations, we identified the risk of temperature-related AMI hospital admissions by gender, age, and living area, and individual-SES.

## 5. Conclusions

Significant increases in AMI risk for several subgroups were associated with temperatures above or below the temperature threshold in both hot and cold weather periods. Our findings indicate that individual SES was one of the important factors affecting for temperature-associated AMI hospital admissions with gender, age, and living area. These findings could help to identify vulnerable groups who are at increased risk for hospital admission due to AMI related to climate change. The data could be used to establish climate change adaptation strategies for susceptible populations.

## Supplementary Materials

**Table S1:** The relative risk (RR) of heat effects on AMI hospital admissions in hot weather period by gender, age, and area.

	Heat Effect ^a^	
	Lag ^b^	RR (95% CI)
**All**	0	1.05 (1.00–1.11) *
**Gender**		
Male	1	1.03 (0.97–1.10)
Female	0	1.10 (1.01–1.20) *
**Age**		
<75 years	0	1.02 (0.96–1.08)
≥75 years	0	1.12 (1.01–1.24) *
**Area**		
Urban	0	1.03 (0.96–1.11)
Rural	0	1.04 (0.99–1.16)

CI: Confidence interval; Models adjusted for calendar year, day of the week, precipitation, humidity, sea-level pressure, and air pollutants (PM_10_, O_3_, NO_2_) as spline functions and considering the annual population size as the offset variable; ^a^ Temperature increase of 1 °C above the threshold maximum temperature (33.5 °C) in hot weather (June–August). Considering duration of heat wave in model; ^b^ Lags of 0, 1, 2–3, 4–7, 8–14, 15–21, 22–28; * *p* < 0.05.

**Table S2:** The relative risk (RR) of cold effects on AMI hospital admissions in cold weather period by gender, age, and area.

	Cold Effect ^a^	
	Lag ^b^	RR (95% CI)
**Total MI**	2–3	1.03 (1.00–1.05) *
**Insurance type**		
NHI	2–3	1.03 (1.01–1.06) *
Medicaid	8–14	1.08 (0.95–1.23)
**Insurance premium**		
Low	22–28	1.05 (1.01–1.10) *
Medium	2–3	1.04 (1.00–1.09)
High	2–3	1.04 (1.00–1.09)

CI: Confidence interval; Models adjusted for calendar year, day of the week, precipitation, humidity, sea-level pressure, and air pollutants (PM_10_, O_3_, NO_2_) as spline functions and considering the annual population size as the offset variable; ^a^ Temperature decrease of 1 °C below the threshold mean temperature (−8.5 °C) in cold weather (December–February). Considering duration of cold wave in model; ^b^ Lags of 0, 1, 2–3, 4–7, 8–14, 15–21, 22–28; * *p* < 0.05.
